# TRI Genotyping and Chemotyping: A Balance of Power

**DOI:** 10.3390/toxins12020064

**Published:** 2020-01-21

**Authors:** Ria T. Villafana, Amanda C. Ramdass, Sephra N. Rampersad

**Affiliations:** Department of Life Sciences, Faculty of Science and Technology, The University of the West Indies, St. Augustine, Trinidad and Tobago

**Keywords:** genotyping, chemotyping, *Fusarium*, molecular detection, trichothecenes

## Abstract

*Fusarium* is among the top 10 most economically important plant pathogens in the world. Trichothecenes are the principal mycotoxins produced as secondary metabolites by select species of *Fusarium* and cause acute and chronic toxicity in animals and humans upon exposure either through consumption and/or contact. There are over 100 trichothecene metabolites and they can occur in a wide range of commodities that form food and feed products. This review discusses strategies to mitigate the risk of mycotoxin production and exposure by examining the *Fusarium*-trichothecene model. Fundamental to mitigation of risk is knowing the identity of the pathogen. As such, a comparison of current, recommended molecular approaches for sequence-based identification of Fusaria is presented, followed by an analysis of the rationale and methods of trichothecene (TRI) genotyping and chemotyping. This type of information confirms the source and nature of risk. While both are powerful tools for informing regulatory decisions, an assessment of the causes of incongruence between TRI genotyping and chemotyping data must be made. Reconciliation of this discordance will map the way forward in terms of optimization of molecular approaches, which includes data validation and sharing in the form of accessible repositories of genomic data and browsers for querying such data.

## 1. Introduction

Mycotoxins are produced by some fungi as toxic secondary metabolites and impose a serious economic impact at all levels of food and feed production, including crop and animal health and production, processing, and distribution and human health [1,2]. These toxic effects can be reversible and irreversible depending on a number of factors [3]. Mycotoxin-producing fungi can be broadly classified into two groups: field fungi (e.g., *Fusarium* species) infect seeds before harvest and produce mycotoxins in the field (pre-harvest infection in seeds with a high moisture content (22 to 25%); and storage fungi (e.g., *Aspergillus*, *Fusarium* and *Penicillium* species) infect stored seeds/grain and produce mycotoxins on stored produce (post-harvest in seeds/grain with 12 to 18% moisture content) (http://www.fao.org). Mycotoxins remain as residues in stored produce within 24 h after fungal infestation [4]. There is, however, overlap between the two designations [3,5].

An effective mycotoxin management program should address prevention of mycotoxin production, detoxification and decontamination, strategies for routine surveillance, implementation of mycotoxin thresholds in contaminated food and feed, and measures to regulate the movement of mycotoxin-contaminated material in national and international trade. In Europe, increasing levels of T-2 and HT-2 Type A trichothecenes in small grain cereals (e.g., wheat, barley, oat, rye, and triticale) is an emerging issue of food safety as these mycotoxins are considered to be high risk due to their common occurrence and high acute toxicity [6,7,8]. Deoxynivalenol (DON) and its acetylated derivatives (3-ADON and 15-ADON) as Type B trichothecenes occur as the predominant mycotoxin in the northern hemisphere and its toxigenic impact is significant to animal health and causes acute human toxicosis [9].

The Food and Agriculture Organization (FAO) and the Codex Alimentarius Commission have adopted a “Hazard Analysis and Critical Control Point (HACCP)” approach, which is a coordinated system that identifies, evaluates, and defines the means by which to control hazards with the potential to cause adverse health effects (http://www.fao.org). Factors that impact upon such regulatory decisions towards mitigating the risk of mycotoxin exposure in human food/feed include but are not limited to:

▪ Identifying the source of mycotoxin contamination, i.e., fungus and toxin identification;
▪ Toxicological profiling of mycotoxin residues in stored food/feed;
▪ Assessing the current analytical methods to identify and quantify such residues;
▪ Defining the relationship between mycotoxin levels and different types of food/feed;
▪ Effects of mycotoxins on human and animal health.

Using the *Fusarium*-trichothecene (TRI) mycotoxin model, at the core of this HACCP system, central to the mitigation of risk of mycotoxin exposure, the following aspects form the basis of this review:

1. Rapid and accurate detection and identification of the *Fusarium* species infecting plant material as an indication of the source of mycotoxin contamination;

2. Identification and quantification of mycotoxins;

3. Toxicological profiling of mycotoxin residues and their metabolites through testing for the presence of the end-products of trichothecene (TRI) biosynthesis, which includes utilization of chemotyping techniques according to their advantages and disadvantages;

4. Potential for prediction of mycotoxin contamination through TRI genotyping, which involves PCR-based methods to detect target genes within the trichothecene biosynthesis gene cluster, and in some cases, assessing the expression levels of TRI genes at the level of the transcript;

5. Characterization of the causes associated with discordance between genotyping and chemotyping data and factors affecting reliability of both approaches to mycotoxin detection.

## 2. Molecular Identification of *Fusarium* Species

Correct identification of *Fusarium* species is fundamental to determining the potential for trichothecene production. The *Fusarium* genus has a membership of 300 phylogenetically distinct species, 20 species complexes and nine monotypic lineages [10,11]. Identification of *Fusarium* to the species level based on morphological characteristics of colony and micro- and macroconidia is prone to error due to the plasticity of morphological traits. Furthermore, not all features needed for identification are well-developed in culture (e.g., the inability of isolates to produce macroconidia after subculture). As such, morphology frequently fails to distinguish among Fusaria at the species level [12].

Multi-locus sequence data comparison is the foundation of current *Fusarium* species identification strategies [11,13,14]. Sequence repositories that house validated protein-coding gene sequences are accessible at *Fusarium* MLST at the CBS-KNAW Fungal Biodiversity Centre (http://www.cbs.knaw.nl/Fusarium/). This database only banks carefully curated sequences of isolates that are available from the CBS-KNAW, *Fusarium* Research Center (FRC, http://plantpath.psu.edu/directory/specialties/Fusarium-research-center) or ARS Culture Collection (NRRL, http://nrrl.ncaur.usda.gov/). Additionally, the accession records that identify a sequence to the phylogenetic species by EF-1a haplotype can be retrieved, e.g., FIESC 25-a, where “25” is the species and “a” is the haplotype within species [15].

The recommended markers for identification of *Fusarium* species are, minimally, the protein-coding genes of the translation elongation factor 1 (*EF-1α*/*TEF-1*/*TEF1*), and the RNA Polymerase II largest and/or second largest subunit (*RPB1* and/or *RPB2*, respectively). The rationale is: (i) sequence comparisons of two independent loci improves the accuracy of identification, (ii) these gene targets are faithfully amplified by PCR and sequenced using primers that are can be successfully used for most members of this genus, (iii) these markers distinguish among sequences at or near species-level, and (iv) these gene sequences are well-represented in the *Fusarium* MLST database [11,16]. Multi-locus sequence typing schemes may include other genetic markers (e.g., Calmodulin—*CAM*, beta- tubulin—*βTUB*) specifically developed for identification of members of defined species complexes. There are also specific primers for the detection of *F. culmorum*, *F. poae, F. sporotrichioides, F. cerealis, F. pseudograminearum, F. graminearum sensu lato,* and *F. graminearum sensu stricto* (Table 1).

Assignment of correct identities to multi-locus sequences involves careful editing of nucleotide sequences to ensure primer sequences are removed, there are no ambiguities in the sequences and the top BLASTn hits match the same species names. Additionally, BLASTx can first be used to verify the identity of the protein-coding regions of the sequences. Where there are several top BLASTn hits with different *Fusarium* species names with similar scores, it is usually necessary to sequence additional loci; however, selection of additional markers excludes the use of ITS and D1/D2 sequences, which cannot resolve sequences to the species level due to low degree of sequence variation [11]. It was also reported that up to 50% of ITS and D1/D2 *Fusarium* sequences curated in GenBank are misidentified, and hypervariable ITS2 sequences of the ITS1-5.8S-ITS2 rDNA array can exist as paralogues or orthologues in several species’ complexes [29,30].

## 3. *Fusarium* Species Known to Produce Trichothecenes

Type A trichothecene (e.g., T-2, HT-2, NEO, DAS) producers, either singly or in co-mixtures, include *F. langsethiae, F. poae, F. polyphialidicum,* and *F. sporotrichioides* (Biomin http://www.mycotoxins.info/mycotoxins/common-mycotoxins/t-2-toxin/) [31,32,33,34]. Type B trichothecene (e.g., NIV, DON) producers are *F. culmorum, F. graminearum, F. poae, F. meridionale, F. sambucinum,* and *F. solani*, some of which are capable of producing one or more trichothecenes of either Type A and/or Type B, e.g., DON in the US [35], DAS, T-2, NEO in Egypt, and NIV, 15-ADON in Argentina [36].

## 4. Chemotyping

The chemotype is defined as the chemical phenotype of a given fungal strain including a profile of the organisms’ secondary metabolites [37]. Chemotyping trichothecene-producing Fusaria is important to determining the potential risk of toxin production in contaminated food and feed, and to devising preventative measures to mitigate this risk. Trichothecenes have been classified into four groups (Types A, B, C, and D) based on the substitution pattern of 12, 13-epoxytrichothec-9-ene [38]. Pasquali et al. [39] described two chemotypes within Type B trichothecenes (Chemotype I and II). Chemotype I which pertains to DON and its acetylated derivative producers, is subdivided into Chemotype IA for 3-ADON producers, and Chemotype IB for 15-ADON producers, while Chemotype II are the NIV and/or 4-ANIV producers. 

### Analytical Techniques for Chemotyping

Ideally, an orthogonal approach to chemotyping is preferred by which the toxin is both quantified and mass-verified. In some cases, a chemical phenotype may relate to a continuum of chemotypes, rather than discrete groupings. The goal is to derive objective classification systems to identify *Fusarium* species based on their chemical profiles. The choice of screening and quantitative methods for the most common Type A and B trichothecenes will depend on the instrumentation available, detection limit required, matrix composition, and the properties of the analyte (Table 2).

## 5. Genotyping 

Genotyping generates TRI gene information pertaining to which taxa are potentially toxic and aids in assigning the toxic share of genotypes in the environment and/or in a given pathogen population. A TRI genotype refers to a specific nucleotide sequence of one or more of the TRI genes found in the genomes of some Fusaria that encodes an enzyme which enables production of a specific trichothecene(s) and, therefore, detection targets the TRI gene sequences. A chemotype is the secondary metabolite profile of an organism as determined by chemical analysis and, therefore, detection targets the end products of trichothecene biosynthesis. A genotype is not a chemotype or vice versa and should not be used interchangeably. As such, on the basis of the proportion of potentially ‘toxic’ genotypes, it should be possible to predict whether there is a risk of toxin production and subsequent exposure [40]. A number of studies have utilized genotyping as a proxy to determining chemotype [41,42,43,44,45,46,47,48,49]. Table 3 outlines the various research efforts in applying TRI genotyping to *Fusarium* species over the last 20 years.

### 5.1. Genotyping Platforms

The development and optimization of molecular tools for detecting genes involved in trichothecene biosynthesis are hinged on an understanding of the arrangement, diversity and evolutionary maintenance of TRI genes as part of a biosynthetic gene cluster [70]. Furthermore, TRI biosynthesis should be characterized as an interconnected network in which various intermediates and a range of end-products are generated; there are alternate routes of synthesis and it is very unlikely the reactions occur as a linear pathway [71].

The commonly used genotyping platforms to investigate the extent and pattern of genetic variation (genotype) that contributes to production of a specific toxin or class of toxin (chemotype) include genotyping-by-sequencing, nucleotide polymorphism detection, quantitative detection of toxins and quantitative detection of toxin-producing *Fusarium* by expression analysis. Detecting and characterizing unique genetic features highlighted by genotyping assays require that the template DNA and RNA be of high integrity regardless of the assay. A genotype-phenotype correlation is not always evident or straightforward due to interactions between different genes, the underlying and often complex biochemical mechanisms of gene expression and the interplay of environmental factors. According to Houle et al. [72], genotype data supplements rather than supplants phenotypic information.

#### 5.1.1. Targeted Detection of TRI Genes by Conventional PCR: Single, Duplex, and Multiplex PCR Assays

One molecular approach to trichothecene genotyping is based on PCR detection and identification of specific TRI genes involved in the trichothecene biosynthesis pathway. For an historical account of the development of primers and PCR conditions for *Fusarium* genotyping see review by Pasquali and Migheli [96]. Specific TRI genes enabled differentiation between different trichothecenes: *TRI3* and *TRI12*, for the differentiation of genotypes into 3-ADON, 15-ADON, or NIV [73,74,78,81,83,91,97] *TRI7* and *TRI13*, for the differentiation of genotypes into DON and NIV [74,81,83,91]; and *TRI1* along with its regulatory genes *TRI6* and *TRI10* [39] and *TRI3* and *TRI12* [83,98], for the differentiation of 3ANX (NX-2) and NX (NX-3) from 3-ADON, 15-ADON, and DON [82]. 

#### 5.1.2. Detection of TRI1 Gene Sequence Polymorphisms by PCR-RFLP

Polymorphisms in the target sequence to be amplified by PCR have been used to differentiate between 3-ADON and NX-2-producers [98]. *TRI1* specific PCR assays are based on amplification of TRI1 gene sequence using a reverse primer (TRI1-R; 5’-TTCCTGCAGGGGCTTGATG-3’) and one of two forward primers for the detection of 3-ADON (5’-AATGCTCGCGAACTAATCAC-3’), and for the detection of 3-ANX (5’AATGCTAGCGAAATGATCAA-3’) genotypes. Polymorphisms in the amplified sequence allows cleavage by *Apo*I restriction enzyme into a specific banding pattern (PCR fingerprinting) that is characteristic of NX-2-producing *F. graminearum* strains, and hence, the NX-2 genotype can be distinguished from Type B genotypes (i.e., 15-ADON, 3-ADON and NIV) of *F. graminearum* [40]. Polymorphisms in the *TRI13* gene sequence is used to distinguish between the DON and the NIV genotypes based on differential size of the amplicon produced: ~227 bp is produced for a DON genotype when primers Tri13F and Tri13DONR are used, whereas, ~312 bp is produced for a NIV genotype by primers Tri13NIVF and Tri13R [99,100,101].

#### 5.1.3. TRI5-TRI6 Intergenic Region Sequencing 

Bakan et al. [102] developed a PCR-based approach for discriminating between high-producing and low-producing *F. culmorum* strains based on amplification with specific primers that target the intergenic region between TRI5 and TRI6 genes (TRI6-54, N1-2, N1-2R, 4056, 3551). PCR amplification with primer pair, N1-2 and N1-2R, resulted in a 200 bp amplicon for the high-producing strains, whereas no amplification was obtained for low-producing strains. PCR amplification with the 4056 and 3551 primers, resulted in an amplicon of 650 bp for the low-producing strains, and no amplification was obtained for high-producing strains. A duplex PCR was carried out with N1-2/N1-2R and the 4056/3551 primer pairs; this enabled differentiation of the high-producing from the low-producing *F. culmorum* strains. 

#### 5.1.4. Multi-locus Genotyping Assay (MLGT) 

A multi-locus genotyping assay (MLGT) was developed to allow simultaneous determination of species identity and trichothecene genotype [103]. Six gene targets, species identification genes (RED, MAT, and TEF-1) and TRI genes (*TRI101*, *TRI12*, *TRI3*), were amplified in a multiplex PCR. PCR products were subjected to allele-specific primer extension (ASPE) reactions in multiplex reactions containing 48 ASPE probes consisting of each species and a type B trichothecene genotype targeted [103,104,105,106]. The resulting biotinylated extension products from ASPE reactions were then hydridized to polystyrene microsphere sets and detection were performed using a Luminex 100 flow cytometer (Luminex Corporation) [103,107].

#### 5.1.5. Quantitation of TRI Gene Products by Real-time qPCR 

qPCR single and multiplex PCR detection can be used to determine genotype profile by directly using the fungal substrate or in food [75,83,108,109,110]. Data from qPCR analysis can be used for qualitative and quantitative analyses in addition to generating gene expression profiles of specific *TRI* genes involved in DON biosynthesis during (i) infection, (ii) colonization, and (iii) according to substrate composition [111,112]. 

## 6. Advantages of TRI Genotyping

The genome sequences of several *F. graminearum* species complex (FGSC) strains have been published. Additionally, the nucleotide sequences of the core trichothecene biosynthetic gene cluster of many representative strains that produce 3-ADON, 15-ADON, and NIV have been deposited in GenBank. This availability of sequence information has enabled the design and selection of several primer sets for molecular characterization of various *Fusarium* strains and species. There are currently 14 complete nucleotide sequences of the trichothecene biosynthetic gene cluster of *F. graminearum* and *F. culmorum* in GenBank. Genotyping requires the design and optimization of primer pairs that target one or more gene of the trichothecene biosynthetic pathway. Availability of several species’ genome sequences have allowed development of the primers. Genotyping also allows screening a large number of isolates in a given fungal population and, therefore, provides the option for high-throughput analysis sample sizes far in excess of what is possible in an analytical chemotype determination in terms of speed and number of samples to process.

The practicality of genotyping is highlighted in the identification of novel chemical groups and *Fusarium* species. Ward et al. [103], through genotyping, confirmed the replacement of a once dominant FHB strain in the US by a more highly toxigenic *F. graminearum* population, which explained the shift in the chemotype composition of *F. graminearum*. A shift in genotype profile may hint at a shift in species [94,99,113,114,115,116,117,118,119]. Newly encountered sequence variation in the specific TRI genes can lead to the production of different chemical end-products which could escape detection by chemical analysis as in the case of NX-2. Monitoring changes in amino acid sequence of TRI genes would enable prediction of a shift in toxin production. The NIV-producing population in Louisiana, USA [106,120], and similarly, a new species in Ethiopia were identified through genotyping [104]. The discovery of novel trichothecene metabolites indicates suggests that the TRI gene markers used for genotyping are integral to genotyping and there may be a need to develop novel and more universal markers for toxin detection [121].

In breeding against *Fusarium* head blight (FHB) susceptibility, it is important to understand the chemotype diversity of the pathogen [122,123,124,125,126]. In fact, assessing the sequence (nucleotide and amino acid) diversity of TRI genes of *Fusarium* strains and their toxin-producing capabilities in breeding programs are considered to be crucial for developing varieties that are more tolerant or resistant to infection [127].

Introduction of novel genotypes into new agroecosystems would challenge *Fusarium* disease management schemes because selection drives the establishment of the more pathogenic fungus. Therefore, for rapid and accurate detection of TRI, genotypes should be considered as an important aspect of quarantine and biosecurity mechanisms [128]. In terms of transnational and international trade, grains produced and exported from NIV-producing populations in different countries should be more closely monitored for toxin contamination.

## 7. Incongruence between Chemotype and Genotype

There is a need for continuous monitoring of *Fusarium* populations at two main tiers: (i) to determine changes in *Fusarium* species, e.g., new introductions, in a given environment [117,129,130] and (ii) to detect shifts in toxin production where a more potent toxin is being produced, e.g., NIV is more toxic than DON, and/or where a toxin is being produced at higher concentrations than previously recorded as a result of species introduction and/or host-environmental adaptation [103,108,114,122,126,131,132,133]. 

Monitoring requires rapid, accurate, and affordable tools to predict toxin production in the field and molecular diagnostic methods can be used as an interim proxy for determining levels of risk. Trichothecene genotyping is a fast and reliable method for the prediction of trichothecene production in various *Fusarium* species based on the identification of specific TRI genes involved in the trichothecene biosynthesis pathway [83]. Some studies reported that genotyping was highly correlated to chemotyping and that trichothecene genotyping enabled rapid prediction of production of different toxins [74,83,90,95,108,134,135]. Other studies report incongruence between chemotype and genotype data and warn against using genotyping to solely identify the risk of mycotoxin production [19,37,82,87,136,137].

There can be incongruence between the TRI genotype and chemotype, which underscores the need for optimized molecular and chemical analytics for the characterization of different toxigenic *Fusarium* species. Additional research into methods to support chemotype determination is required as the currently available chemotyping techniques are dependent on induction of trichothecene production by isolates in vitro. The chemotype profile in the field and the profile determined in the laboratory are at the very least partially discordant, and for which, for various reasons (some of them still undefined), chemotype data may fail to predict the “real” risk of toxin production by select *Fusarium* strains. Genotyping provides baseline data for evaluating mycotoxin risk, as this approach confirms the presence of TRI genes in a given genome [138]. Kelly et al. [84] stated that it is possible to accurately infer chemotype from trichothecene genotype based on the NX-2 Type B trichothecene case study. The challenge lies in the induction of mycotoxins in vitro for subsequent chemical detection and quantification.

## 8. Factors Affecting the Reliability of Genotype-chemotype Association

In vitro toxin induction, production and concentration are strain-, substrate-, temperature-, pH-, water activity-/relative humidity-, and time-dependent for many *Fusarium* species [139]. In addition, the expression of TRI genes that lead to synthesis of trichothecenes and their metabolites or acetylated derivatives is not a linear pathway and should be considered to be a network with multiple routes to a given toxin [71]. This expression is subject to a number of factors that are as yet undefined [37,140]. 

In vitro induction of trichothecenes for subsequent chemical determination of chemotype minimally necessitates that media (composition of substrate) and incubation conditions are considered for maximum mycotoxin induction. There are no defined conditions for toxin induction for several *Fusarium* species, excluding *F. graminearum*; however, research has shown that even within a given species, different strains within the same geographical location can behave differently under laboratory conditions [39,96,141,142,143,144]. Furthermore, although there are reports of protocols for the induction of trichothecenes by *F. graminearum* in vitro based on liquid cultures [145,146,147,148,149,150], induction of trichothecene production under laboratory conditions remains the limiting factor in detection. Genotyping of *F. graminearum* sensu stricto strains infecting wheat in Minnesota resulted in 3-ADON genotype, but chemical analysis indicated that neither DON nor NIV nor its acetylated derivatives were produced in vitro [151]. The danger in this case was these ‘no trichothecene’ producers were then studied as potential biocontrol agents and the failure to induce trichothecene in vitro and the resultant failure of the chemical analytical method to detect any toxin were not considered [152]. Sugiara et al. [153] also pointed out that NIV-producing strains can also produce low levels of DON, but DON-producers cannot produce NIV. Chemical analysis has to cater to the different options to produce trichothecenes by these strains.

One possible explanation for the similar accumulation of both acetyl derivatives by strains of different chemotype and genotypes could be that the acetyl derivatives biosynthesis (DON) is regulated by temperature [39,154,155]. Temperature influences trichothecene chemotypes [39,154,155]. At high temperatures (above 30 °C), 3-ADON production is favored, with minimum production of 15-ADON between 30–35 °C, while at low temperatures (below 10 °C) 15-ADON production is favored, with minimum production of 3-ADON between 5–10 °C [39,154,156,157]. Variations in the respective concentrations can also be expected for each chemotype with trace amounts of 15-ADON being produced for Chemotype 1A (between 30–35 °C) and vice versa for Chemotype 1B between 5–10 °C. In the case of Chemotype B (NIV and its derivatives) producers, changes in climate had no effect on the production of this mycotoxin [156,158]. 

Most studies that focused on the effects of abiotic factors on mycotoxin production examined the effects of temperature and incubation time, but few have looked at the effects of water activity *aw* on mycotoxin production [144,159]. Hope et al. [160] determined that the minimum *aw* required for DON production to occur in *F. graminearum* and *F. culmorum* is > 0.93 *aw* at optimum temperature; however, below 0.90 *aw*, no DON is produced. In another study, maximum DON production was observed at 0.995 *aw* at 25 °C, over a 40 days period, on wheat-based media, but as the water availability decreased, DON production also decreased at a significant rate. At lower temperatures (15 °C), maximum DON production was only observed at > 0.981 *aw*. The study also looked at the effects of water availability and NIV production under the same conditions, specifically at 0.995 *aw* and 25 °C, NIV production was 10 times less than DON over a 40-day period but was greater at 15 °C, with maximum NIV production occurring at 0.981 *aw* [161].

Simultaneous production of different toxins can often be missed by analytical chemotyping methods where testing is for one target toxin. Different toxins (e.g., NIV and DON) can be simultaneously produced by the same isolate depending on induction conditions [86,162]. Chemical analysis would also have to cover detection of acetylated and non-acetylated forms of DON as several studies reported the co-production of acetylated forms of trichothecenes by certain strains (3-ADON and 15-ADON), albeit in different relative amounts [86,163,164,165]. New toxins would escape detection by chemotyping methods due to a lack of standards for a new toxin [166].

The quantitative toxin production capacity of individual *Fusarium* strains can vary significantly, indicating that strains isolated from the same geographical region may have different abilities to produce toxins and produce them in differing levels in vitro [167]. Toxin production is often variable among isolates and some strains simply do not produce any toxins under laboratory conditions [136,168,169].

Conditions that regulate toxin production in the field are impacted by complex environment-plant-pathogen interactions and toxin production in vitro may not reveal the toxigenic potential of a given strain, which can be used to devise strategies to mitigate risk of exposure [170,171,172]. In planta field inoculation may be able to demonstrate the toxigenic capability of *Fusarium* strain, but even so, there is a myriad of factors that influence toxin production under field conditions [173,174]. The interrelated conditions that simulate the toxin-producing behavior of a strain in the field are not defined. For instance, fungicide use in a given agroecosystem may have an effect on the chemotype of the *Fusarium* population in that environment [175]. Specifically, carbendazim (MBC) resistance can be associated with higher toxin production [176]. The 3-ADON chemotype in Asia is predominant in *F. graminearum* and *F. asiaticum* populations [101]. According to Zhang et al. [177], the 3-ADON chemotype had an adaptive advantage over NIV-producing *F. asiaticum* strains, in that they were more resistant to benzimidazoles. However, these data were relevant to a specific *Fusarium* population and the history and conditions under which MBC was utilized must be considered. Carbendazim-resistant isolates produced higher concentrations of trichothecenes (DON+3-ADON or DON+15-ADON) than carbendazim-S isolates in vitro and in field inoculated wheat heads [178]. Kulik et al. [77] carried out mycotoxin analysis of three *F. graminearum* isolates of 3-ADON, 15-ADON, and NIV chemotypes using RT-qPCR and the results indicated an increase in trichothecene accumulation in most of the tebuconazole-treated samples. 

The host plant also plays a defining role in determining the toxicity of a toxin and specific examples are: (i) the ability of potato host to transform DON into NIV [35], (ii) NX-2 is detoxified to non-toxic rearrangement products (NX-3 and NX-3-M1) in planta [166], and (iii) the ability of resistant wheat genotypes to metabolize DON [174,179].

Trichothecenes also function as virulence factors for different *Fusarium* head blight (FHB) pathogens [39,180,181,182]. As such, different *Fusarium* species have been shown to exhibit host preference and different levels of competitiveness in cases of co-infection [183]. In China, maize is more susceptible to infection by *F. asiaticum* (Chemotype II) and is generally more aggressive than other Chemotype I-producing *Fusarium* sp. [39,99,154,184]. On the other hand, in wheat and rice, Chemotype IA-producers, e.g., *F. asiaticum* are more aggressive than Chemotype II producers and produce higher concentrations of DON [101,155].

## 9. Future Prospects—Data Sharing and Quality Control

Concerns have been raised about sequence identities in GenBank as the largest and most widely used database. Apart from the discrepancy in using ITS sequences alone for sequence identification, some entries lack descriptive and up-to-date annotations due to the rapid pace of fungal taxonomy revision, type strains may not be clearly indicated, sequences may be unnamed or only partially named, and there are unpublished sequences in GenBank, which is an indication of authenticity to many researchers [185].

O’Donnell et al. [11] published clear guidelines and a 10-step primer for obtaining accurate species-level identification based on BLASTn queries. The study described sequence-based identification of Fusaria using databases that contain a library of sequences from type strains and whose sequence identity has been verified, i.e., the CBS-KNAW Biodiversity Centre (http://www.cbs.knaw.nl/Fusarium/). The reporting standards in *Fusarium* MLST ensure that (i) submitted data are sufficient for clear interpretation and querying by other researchers, (ii) data formats are standardized, (iii) terminology used is consistent, (iv) TEF1, RPB2, and RPB1 sequences specific for *Fusarium* identification are verified, (v) haplotype data based on TEF1 analysis is consistent, and finally, (vi) there is clear and accessible information for contacting the specific curators dealing with *Fusarium*-related matters and software/website-related matters.

### 9.1. Data Repositories for Fusarium Genome Sequences

**Figure 1:**
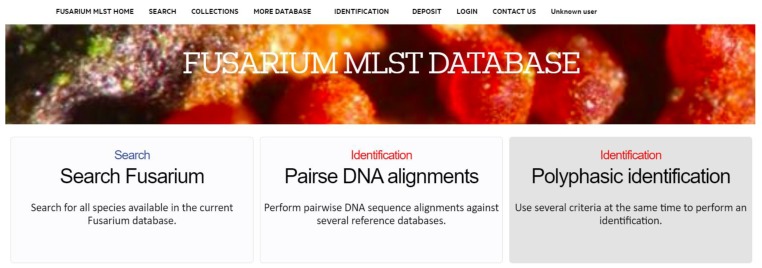
*Fusarium* MLST (http://www.wi.knaw.nl/Fusarium/).

**Figure 2:**
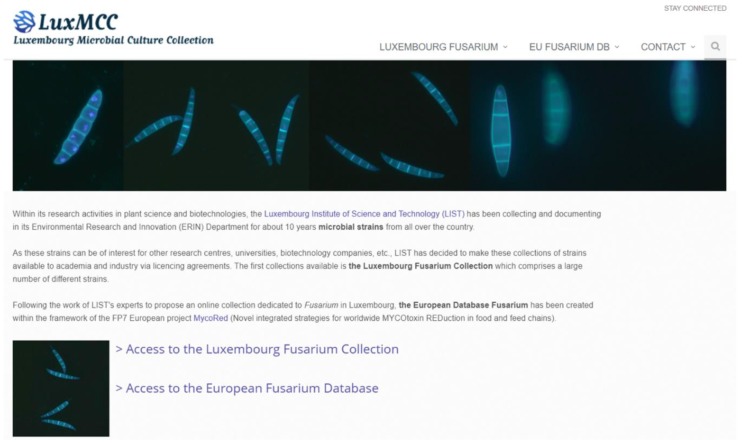
Luxemberg *Fusarium* collection and the European *Fusarium* collection (https://catalogue.luxmcc.lu/).

**Figure 3:**
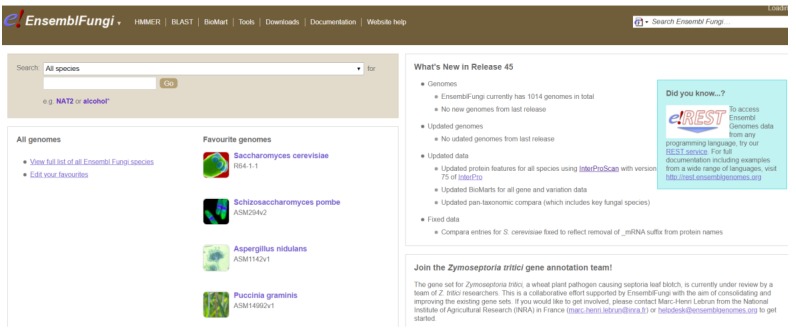
EnsemblFungi (https://fungi.ensembl.org/index.html).

**Figure 4:**
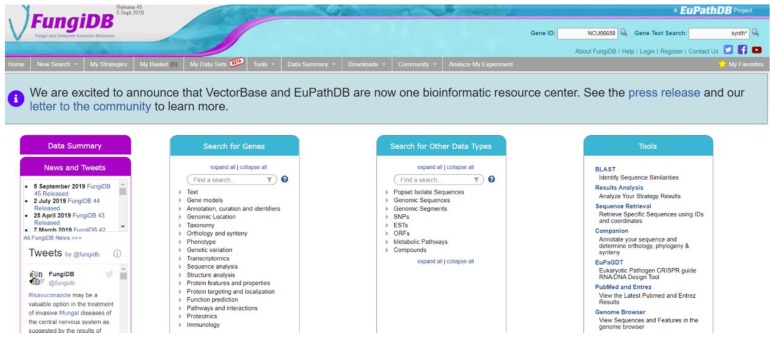
FungiDB (https://fungidb.org/fungidb/).

**Figure 5:**
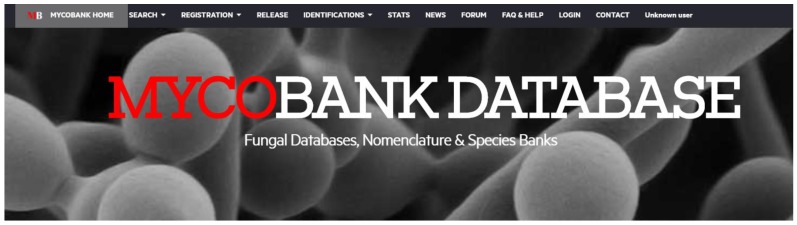
MycoBank (http://www.mycobank.org/).

One of the challenges of developing a genotype database is the availability of molecular as well as other metadata in relation to identification of *Fusarium* strains. There are several repositories, outside of GenBank, that house searchable genomic data for a range of *Fusarium* species. Screenshots of the home page of each of these databases and/or searchable browsers are presented as figures (Figure 1, Figure 2, Figure 3, Figure 4 and Figure 5) under each repository as proof of recent activity and on-going curation. All websites were accessed on 25th October, 2019.

### 9.2. Fusarium MLST 

This database can be queried against for DNA sequence-based identification of single and multiple sequences. The single sequence alignment algorithm compares the sequence of an unknown against sequences present in the *Fusarium* MLST reference database. The multiple sequences option, sequences from two or more loci from the unknown are queried against the *Fusarium* MLST database using tools within the BioloMICS software.

### 9.3. A European Database of F. graminearum and F. culmorum Trichothecene Genotypes

This is a freely accessible and updatable database of trichothecene genotypes of strains from three *Fusarium* species, collected over the period 2000–2013: 1147 *F. graminearum*, 479 *F. culmorum*, and 3 *F. cortaderiae* strains from 17 European countries in addition to mapping of trichothecene type B genotype with respect to distribution according to species [39]. Information on host plant, country of origin, sampling location, year of sampling, and previous crop are available. This information is important to epidemiological analysis of potential spatial and temporal trichothecene genotype shifts in Europe. 

### 9.4. Ensembl Fungi 

Ensembl Fungi is a browser used for exploring fungal genomes. The genome sequences are accessed from the databases of the International Nucleotide Sequence Database Collaboration (the European Nucleotide Archive at the EBI, GenBank at the NCBI, and the DNA Database of Japan).

### 9.5. FungiDB 

FungiDB belongs to the EuPathDB suite of databases and offers an integrated genomic and functional genomic database for fungi. FungiDB also includes experimental and environmental isolate sequence data, comparative genomics, analysis of gene expression, supplemental bioinformatics analyses, and a web interface for data-mining.

### 9.6. MycoBank

This database offers up-to-date taxonomic features and nomenclature, species descriptions, and illustrations in addition to metrics that track these changes. Pairwise sequence alignments of fungi and yeasts against curated references databases are enabled [186,187].

## Figures and Tables

**Table 1:** – PCR primers for the detection of *Fusarium* species as known trichothecene mycotoxin producers.

*Fusarium* Species	Primer Name	Target Gene		Primer Sequence/5’ to 3’	Amplicon/bp	Reference
*F. culmorum*	FC01F (fwd)	SCAR	specific	ATGGTGAACTCGTCGTGGC	570	[17,18]
	FC01R (rev)	SCAR		CCCTTCTTACGCCAATCTCG		
	Fcg17F (fwd)	SCAR	*F. culmorum + F. graminearum*	TCGATATACCGTGCGATTTCC	340	
	Fcg17R (rev)	SCAR		TACAGACACCGTCAGGGGG		
	Fcu-F (fwd)	IGS	specific	GACTATCATTATGCTTGCGAGAG	200	
	Fgc-R (rev)	IGS		CTCTCATATACCCTCCG		
*F. graminearum* + fungi belonging to FGSC - *F. asiaticum; F. meridionale*	Fg16F (fwd)	SCAR	FGSC members	CTCCGGATATGTTGCGTCAA	400–500	[18,19,20]
	Fg16R (rev)	SCAR		GGTAGGTATCCGACATGGCAA		
	Fgr-F (fwd)	IGS	specific	GTTGATGGGTAAAAGTGTG	500	[21]
	Fgc-R (rev)	IGS		CTCTCATATACCCTCCG		
	GOFW (fwd)	*gaoA* gene	specific	ACCTCTGTTGTTCTTCCAGACGG	472	[22]
	GORV (rev)	*gaoA* gene		CTGGTCAGTATTAACCGTGTGTG		
*F. poae*	FP82F (fwd)	SCAR	specific	CAAGCAAACAGGCTCTTCACC	220	[23]
	FP82R (rev)	SCAR		TGTTCCACCTCAGTGACAGGTT		
	PoaeIGS-R (fwd)	IGS	*F. poae + F. kyushuense* + *F. langsethiae*	CAAGCTCTCCTCGGAGAGTCGAA	306	[24]
	CNL12 (rev)	IGS		CTGAACGCCTCTAAGTCAG		
	Fps-F (fwd)	IGS	specific	CGCACGTATAGATGGACAAG		
	Fpo-R (rev)	IGS		CAGCGCACCCCTCAGAGC	400	
*F. sporotrichioides*	AF330109CF (fwd)	TRI13	specific	AAAAGCCCAAATTGCTGATG	332	[25]
	AF330109CR (rev)	TRI13		TGGCATGTTCATTGTCACCT		
	FspITS2K (fwd)	ITS	specific	CTTGGTGTTGGGATCTGTCTGCAA	288	[26]
	P28SL (rev)	ITS		ACAAATTACAACTCGGGCCCGAGA		
	Fps-F (fwd)	IGS	specific	CGCACGTATAGATGGACAAG	400	[21]
	Fsp-R (rev)	IGS		GTCAGAAGAGACGCATCCGCC		
*F. pseudograminearum*	FP1-1 (fwd)		degenerate	CGGGGTAGTTTCACATTTCYG	523	[27]
	FP1-2 (rev)			GAGAATGTGATGASGACAATA		
*F. cerealis*	CRO-AF (fwd)		specific	CTCAGTGTCCACCGCGTTGCGTAG	842	[28]
	CRO-AR (rev)			CTCAGTGTCCCATCAAATAGTCC		

**Table 2:** Current analytical techniques for TRI chemotyping.

Technique	Advantages	Disadvantages	TRI Toxin	References
**Enzyme-Linked Immunosorbent Assay (ELISA)**	(1) several ELISA-kits capable of detecting DON in the relevant concentration range set by the FDA and EU are commercially available; (2) analysis of several samples in a single test - high throughput and portability for on-site application; (3) simple sample processing; (4) high sensitivity and specificity; (5) does not require toxic reagents; (6) can detect the presence of fungi in food even after heat treatment which enables the evaluation of contamination in processed foods; (7) rapid screening (<0.5-2h); (8) in situ use; (9) test kits available for use with low sample volume requirements and less clean-up steps compared to methods like TLC and HPLC; (10) simultaneous analysis of multiple samples	(1) cross-reactivity and dependence on a specific matrix - cross-reactivity data for 3-ADON, 15-ADON and/or DON-3G were reported in some studies using different commercially available ELISA-kits; (2) matrix interference (presence of other substances lead to alteration of results); (3) semi-quantitative method and confirmatory reference method is required; (4) narrow operating range; (5) false positive/negative results possible; (6) each kit detects only a single mycotoxin and is designed for one-time use; thus, it can be costly if one needs to test samples contaminated with multiple mycotoxins; (7) each test kit is specified by the manufacturer and while some third-party validations, e.g., by AOAC, have been done for some mycotoxin ELISA kits, the validation and marketing are for use with specific toxins under specific contamination levels within specified matrixes and, therefore, the kit cannot be used for all food matrices and all contamination levels	DON, T-2, T-2/HT-2	[50,51,52,53,54,55,56,57]
**Liquid chromatography-mass spectrometry (LC-MS) or tandem mass spectrometry (LC-MS/MS); LC-MS/MS followed by structure confirmation via Q-TOF LC/MS, 1H- and 13C-NMR; LC-MS-based methods—LC with efficient electrospray (ESI) or atmospheric pressure chemical ionization (APCI); Columns: DON-NIV™ WB immunoaffinity columns isolate DON and NIV simultaneously in a single sample extract; Myco 6-in-1 is a quantitative method for the simultaneous detection of six mycotoxins**	(1) selective detection; (2) low detection limits; (3) qualitative and quantitative results; (4) generation of structural information of analyte; (5) little sample treatment required; (6) applicable to complex matrices; (7) multi-analyte analysis; (8) no derivatization required	(1) expensive technology—high-end instrumentation to achieve suitable detection limits; (2) specialist expertise required to perform analysis; (3) time consuming when compared to rapid test; (4) sensitivity is dependent on ionization technique-challenge to achieve tight chromatographic conditions (especially pH and additives to the mobile phase) for optimal ionization; (5) optimal ionization only achievable in modern instruments with rapid switching between negative and positive modes as mycotoxins vary, e.g., polarity, molecular mass, and heavy reliance on correct sample preparation and purification; (6) reliable quantification achievable only by matrix-matched calibration and internal standards; (7) matrix-matched calibration to improve performances; (8) may require different extraction solvents, types of clean-up (solid phase extraction (SPE), QuEChERS, and immunoaffinity column (IAC)) as well as calibration approaches (external or matrix matched)	NIV, DON, 3-Ac-DON, 15-Ac-DON, HT-2, T-2 toxin (maize); LC-ESI-MS/MS: NIV, DON, 3-Ac-DON, 15-Ac-DON, HT-2 toxin, T-2 toxin, DAS, neosolaniol, monoacetoxyscirpenol, T-2 triol, and T-2 tetraol (wheat and oat); LC-APCI-MS- DOM-1, HT-2 toxin, T-2 toxin, acetyl T-2 toxin, DAS, monoacetoxyscirpenol, neosolaniol (oats, maize, barley and wheat); T-2 and HT-2 and their glucosylated and acetylated derivatives (T2 toxin-3-glucoside, 3-acetyl-T-2 toxin and 3-acetyl-HT-2 toxin) in staple flours, barley, maize, oats, rye, and wheat	[58,59,60,61,62,63]
**High Performance Liquid Chromatography (HPLC); Columns: T-2/HT-2™ HPLC columns -T-2 and HT-2 toxins**	(1) high sensitivity and selectivity; (2) applicable to complex matrices; (3) high reliability and accuracy; (4) short analysis time; (5) automated (auto-sampler)	(1) expensive technology; (2) laborious; (3) require the use toxic chemicals and there is a cost attributed to waste storage and disposal; (4) specialist expertise required to perform analysis; (5) time consuming when compared to rapid test; (6) compounds must possess UV absorption or fluorescence properties; (7) derivatization may be required	HT-2 toxin, T-2 toxin, DON (cereals and grains)	[58,59,60,64]
**HPLC with a specific detector—fluorescence (FL), ultraviolet (UV), diode array (DAD), or MS; Ultra HPLC-MS/MS (UHPLC-MS/MS)**	HPLC-FL- highly specific and sensitive, lower cost than LC-MS methods; method validation performed according to Commission Decision 2002/657/EC (EC, 2002, 2014, 2017), revealed precision	HPLC-FL—specificity for fluorescing compounds which must be well separated on column for reliable quantification	HPLC-FL- DON, NIV, T-2 toxin, HT-2 toxin, NEO, DAS, 3-Ac-DON, 15-Ac-DON (wheat and corn); HPLC-MS- DON; DON and its acetylated and glucosylated metabolites, HT-2 and T-2 toxins in maize	[62,65,66,67]
**Combination of immunological capture and HPLC-MS/MS**	monoclonal antibody developed against DON for purification of cereal extract, before the follow-up HPLC-MS/MS analysis	N/A	DON, 3-ADON, and 15-ADON from wheat, oatmeal, and maize	[68]
**Competitive immunochromatographic assay or lateral flow immunoassay**	N/A	N/A	DON in maize extracts	[69]

**Table 3:** TRI genotyping of *Fusarium* species over the last 20 years.

*Fusarium* Species	Host Species	Country	TRI Gene Target	Chemotype	Reference
*F. asiaticum*	*Triticum* sp. (wheat)	China	*TRI3, TRI12*	3-ADON	[73]
*F. asiaticum*	*Hordeum vulgare* (barley)	Japan	*TRI3, TRI12*	NIV	[73]
*F. asiaticum*	*Triticum* sp. (wheat)	Taiwan	*TRI13*	15-ADON and NIV	[74]
*F. austroamericanum*	*Zea mays* (maize)	Brazil	*TRI3, TRI12*	3-ADON	[73]
*F. austroamericanum*	herbaceous vine	Venezuela	*TRI3, TRI12*	NIV	[73]
*F. cerealis*	potato tuber	Netherlands	*TRI3, TRI12*	NIV	[75]
*F. cerealis*	Azalea	New Zeland	*TRI3, TRI12*	NIV	[75]
*F. culmorum*	*Ammophila arenaria* (European beachgrass)	Netherlands	*TRI3, TRI12*	NIV	[75]
*F. culmorum*	*Triticum* sp. (wheat)	France	*TRI3, TRI12*	3-ADON	[75]
*F. culmorum*	*Populus nigra* (European black poplar)	Portugal	*TRI3, TRI12*	3-ADON	[75]
*F. culmorum*	*Ammophila arenaria* (European beachgrass)	Netherlands	*TRI3, TRI12*	NIV	[75]
*F. culmorum*	*Hordeum vulgare* (barley)	Denmark	*TRI3, TRI12*	3-ADON	[73]
*F. culmorum*	*Avena sativa* (oat)	Canada	*TRI3, TRI12*	3-ADON	[76]
*F. culmorum*	*Hyacinthus orientalis* (Hyacinth)	Netherlands	*TRI3, TRI12*	NIV	[75]
*F. culmorum*	*Triticum* sp. (wheat)	Poland	*TRI3, TRI12*	NIV and 3-ADON	[77]
*F. culmorum*	*Triticum* sp. (wheat)	UK	*TRI3, TRI7, TRI13*	DON and NIV	[78]
*F. culmorum*	*Triticum* sp. (wheat)	Tunisia	*TRI3, TRI5, TRI7, TRI13*	DON, NIV	[79]
*F. graminearum*	*Zea mays* (maize)	Iran	*TRI3, TRI12*	NIV	[73]
*F. graminearum*	*Triticum* sp. (wheat)	South Africa	*TRI3, TRI12*	15-ADON	[75]
*F. graminearum*	*Rumohra adiantiformis* (leatherleaf fern)	Netherlands	*TRI3, TRI12*	NIV	[73]
*F. graminearum*	*Triticum* sp. (Louisiana, wheat)	USA	*TRI3, TRI12*	15-ADON	[80]
*F. graminearum*	*Triticum* sp. (Ohio, wheat)	USA	*TRI3, TRI12*	3-ADON	[75]
*F. graminearum*	*Zea mays* (Michigan, maize)	USA	*TRI3, TRI12*	15-ADON	[73]
*F. graminearum*	*Zea mays* (Ohio, maize)	USA	*TRI3, TRI12*	15-ADON	[73]
*F. graminearum*	*Triticum* sp. (Kansas, wheat)	USA	*TRI3, TRI12*	15-ADON	[73]
*F. graminearum*	*Sorghum bicolor* (sorghum)	Ethiopia	*TRI3, TRI12*	15-ADON	[75]
*F. graminearum*	*Zea mays* (maize)	Nepal	*TRI3, TRI12*	NIV	[73]
*F. graminearum*	*Avena sativa* (oat)	Sweden	*TRI3, TRI12*	3-ADON	[75]
*F. graminearum*	*Zea mays* (maize)	South Africa	*TRI3, TRI12*	15-ADON	[75]
*F. graminearum*	*Triticum* sp. (wheat)	England	*TRI3, TRI12*	15-ADON	[75]
*F. graminearum*	*Triticum* sp. (wheat)	Poland	*TRI3, TRI12*	3-ADON, 15-ADON and NIV	[75]
*F. graminearum*	*Zea mays* (maize)	Korea	*TRI3, TRI4, TRI5, TRI7, TRI8, TRI11*	DON and NIV	[81]
*F. graminearum*	*Hordeum vulgare* (barley)	Korea	*TRI3, TRI4, TRI5, TRI7, TRI8, TRI11*	DON and NIV	[81]
*F. graminearum*	*Triticum* sp. (wheat)	Taiwan	*TRI13*	15-ADON and NIV	[74]
*F. graminearum*	*Triticum* sp. (wheat)	Canada	*TRI1, TRI8, TRI12, TRI3*	3-ADON, 15-ADON and 3-ANX	[82]
*F. graminearum*	*Zea mays* (maize)	Canada	*TRI1, TRI8, TRI12, TRI3*	3-ADON, 15-ADON and 3-ANX	[82]
*F. graminearum*	*Triticum* sp. (wheat)	Brazil	*TRI3, TRI12*	15-ADON, NIV and 3-ADON	[83]
*F. graminearum*	potato tuber	USA	*TRI7, TRI13*	DON, NIV	[35]
*F. graminearum*	*Triticum* sp. (wheat)	Canada	*TRI1*	DON, NIV, NX-2	[84]
*F. graminearum*	Multiple	USA	*TRI1*	NX-2	[85]
*F. graminearum*	*Galium aparine, Triticum* sp. *Zea mays*	Germany, France	*TRI7, TRI13, TRI5-TRI6*	3-ADON, 15-ADON, DON, NIV	[86]
*F. graminearum + 21 related species of the F. sambucinum s.c.*	cereals	USA (north) + Canada (south)	*TRI1*	NX-2	[40]
*F. graminearum s.c.*	*Zea mays* (maize)	Argentina	*TRI7, TRI13*	DON, NIV	[87]
*F. graminearum s.c.*	*Triticum* sp. (wheat), wild grass	USA (New York)	*TRI1*	NX-2	[88]
*F. graminearum s.c. F. boothii, F. asiaticum, F. meridionale*	*Zea mays* (maize)	Nepal	*TRI13*	DON, NIV	[89]
*F. graminearum s.c. F. pseudograminearum, and F. poae*	*Hordeum vulgare* (barley)	Uraguay	*TRI1*	15-ADON, NX-2	[17]
*F. graminearum s.s.*	*Triticum* sp. (wheat)	Argentina	*TRI3, TRI7, TRI13*	15-ADON, DON, NIV	[90]
*F. graminearum s.s.*	*Triticum* sp. (wheat)	Argentina	*TRI3, TRI7, TRI13*	3-ADON, 15-ADON, DON, NIV	[19]
*F. graminearum s.s.*	*Triticum* sp. (wheat)	Italy	*TRI5, TRI7, TRI3*	NIV	[91]
*F. graminearum s.s.*	*Triticum* sp. (wheat)	Uraguay	*TRI3, TRI5, TRI7, TRI13*	15-ADON, DON	[92]
*F. graminearum s.s. F. culmorum, F. poae*	*Triticum* sp. (wheat)	Italy	*TRI5, TRI7, TRI12*	3-ADON, 15-ADON, NIV	[20]
*F. graminearum s.s. F. meridionale*	*Glycine max* (soybean)	Argentina	*TRI3, TRI5, TRI7*	15-ADON, DON, NIV	[93]
*F. graminearum s.s. F. meridionale, F. austroamericanum*	*Triticum* sp. (wheat)	Brazil	*TRI3, TRI12*	3-ADON, 15-ADON, NIV	[94]
*F. graminearum, F. meridionale*	*Triticum* sp. (wheat)	Brazil	*TRI3, TRI12, TRI13*	15-ADON, DON	[95]
*F. meridionale*	orange twig	New Caledonia	*TRI3, TRI12*	NIV	[73]
*F. meridionale*	*Zea mays* (maize)	Nepal	*TRI3, TRI12*	NIV	[73]
*F. meridionale*	*Triticum* sp. (wheat)	Taiwan	*TRI13*	NIV	[74]
*F. mesoamericanum*	*Musa sp*. (banana)	Honduras	*TRI3, TRI12*	NIV	[73]
*F. mesoamericanum*	*Acaciae mearnsii* (black wattle)	South Africa	*TRI3, TRI12*	NIV	[75]
*F. pseudograminearum*	*Hordeum vulgare* (barley)	Australia	*TRI3, TRI12*	3-ADON	[73]
*F. sporotrichioides*	*Zea mays* (maize)	Korea	*TRI3, TRI4, TRI5, TRI8, TRI11*	NIV	[81]
*F. sporotrichioides*	*Hordeum vulgare* (barley)	Korea	*TRI3, TRI4, TRI5, TRI8, TRI11*	NIV	[81]
